# Left atrial appendage closure in patients with a reduced left ventricular ejection fraction: results from the multicenter German LAARGE registry

**DOI:** 10.1007/s00392-020-01627-8

**Published:** 2020-03-31

**Authors:** Christian Fastner, Johannes Brachmann, Thorsten Lewalter, Uwe Zeymer, Horst Sievert, Martin Borggrefe, Christian Weiß, Volker Geist, Alexander Krapivsky, Matthias Käunicke, Harald Mudra, Matthias Hochadel, Steffen Schneider, Jochen Senges, Ibrahim Akin

**Affiliations:** 1 First Department of Medicine, University Medical Centre Mannheim (UMM), Faculty of Medicine Mannheim, University of Heidelberg, Theodor-Kutzer-Ufer 1-3, 68167 Mannheim, Germany; 2 European Center for AngioScience (ECAS), Mannheim, Germany; 3 DZHK (German Center for Cardiovascular Research) Partner Site Heidelberg/Mannheim, Mannheim, Germany; 4 Department of Cardiology, Angiology, and Pneumology, Second Medical Clinic, Coburg Hospital, Coburg, Germany; 5 Department of Medicine, Cardiology, and Intensive Care, Hospital Munich-Thalkirchen, Munich, Germany; 6 Klinikum Ludwigshafen, Ludwigshafen am Rhein, Germany; 7 CardioVascular Center (CVC) Frankfurt, Frankfurt, Germany; 8 Anglia Ruskin University, Chelmsford, UK; 9 Department of Cardiology, Klinikum Lüneburg, Lüneburg, Germany; 10 Department of Cardiology, Heart Center, Segeberger Kliniken, Bad Segeberg, Germany; 11 Department of Cardiology, Evangelisches Krankenhaus, Mülheim (Ruhr), Germany; 12 Department of Cardiology, University of Witten/Herdecke, Katholisches Klinikum Essen, Essen, Germany; 13 Department of Cardiology, Klinikum Neuperlach, Munich, Germany; 14 Stiftung Institut für Herzinfarktforschung, Ludwigshafen am Rhein, Germany

**Keywords:** Atrial fibrillation, Ischemic stroke, Bleeding risk, LAA closure, Depressed left ventricular function, LAARGE

## Abstract

**Background:**

Interventional left atrial appendage closure (LAAC) effectively prevents thromboembolic events in atrial fibrillation patients. Impaired left ventricular ejection fraction (LVEF) increases not only the thromboembolic risk but also the complication rates of cardiac interventions. The LAAC procedure’s benefit in patients with an impaired LVEF, therefore, has yet to be investigated.

**Methods:**

LAARGE is a prospective, non-randomized registry depicting the clinical reality of LAAC in Germany. Procedure was conducted with different standard commercial devices, and follow-up period was one year. In the sense of an as-treated analysis, patients with started procedure and documented LVEF were selected from the whole database.

**Results:**

619 patients from 37 centers were categorized into one of three groups: LVEF > 55% (56%), 36–55% (36%), and ≤ 35% (8%). Prevalence of cardiovascular comorbidity increased with LVEF reduction (*p* < 0.001 for trend). CHA_2_DS_2_-VASc score was 4.3, 4.8, and 5.1 (*p* < 0.001), and HAS-BLED score was 3.7, 4.1, and 4.2 (*p* < 0.001). Implantation success was consistently high (97.9%), rates of intra-hospital MACCE (0.5%), and other major complications (4.2%) were low (each *p* = NS). Kaplan–Meier estimation showed a decrease in survival free of stroke with LVEF reduction during one-year follow-up (89.3 vs. 87.0 vs. 79.8%; *p* = 0.067), a trend which was no longer evident after adjustment for relevant confounding factors. Rates of non-fatal strokes (0.4 vs. 1.1 vs. 0%) and severe bleedings (0.7 vs. 0.0 vs. 3.1%) were consistently low across all groups (each *p* = NS).

**Conclusions:**

LVEF reduction neither influenced the procedural success nor the effectiveness and safety of stroke prevention by LAAC.

**Trial Registration:**

ClinicalTrials.gov Identifier: NCT02230748

**Graphic abstract:**

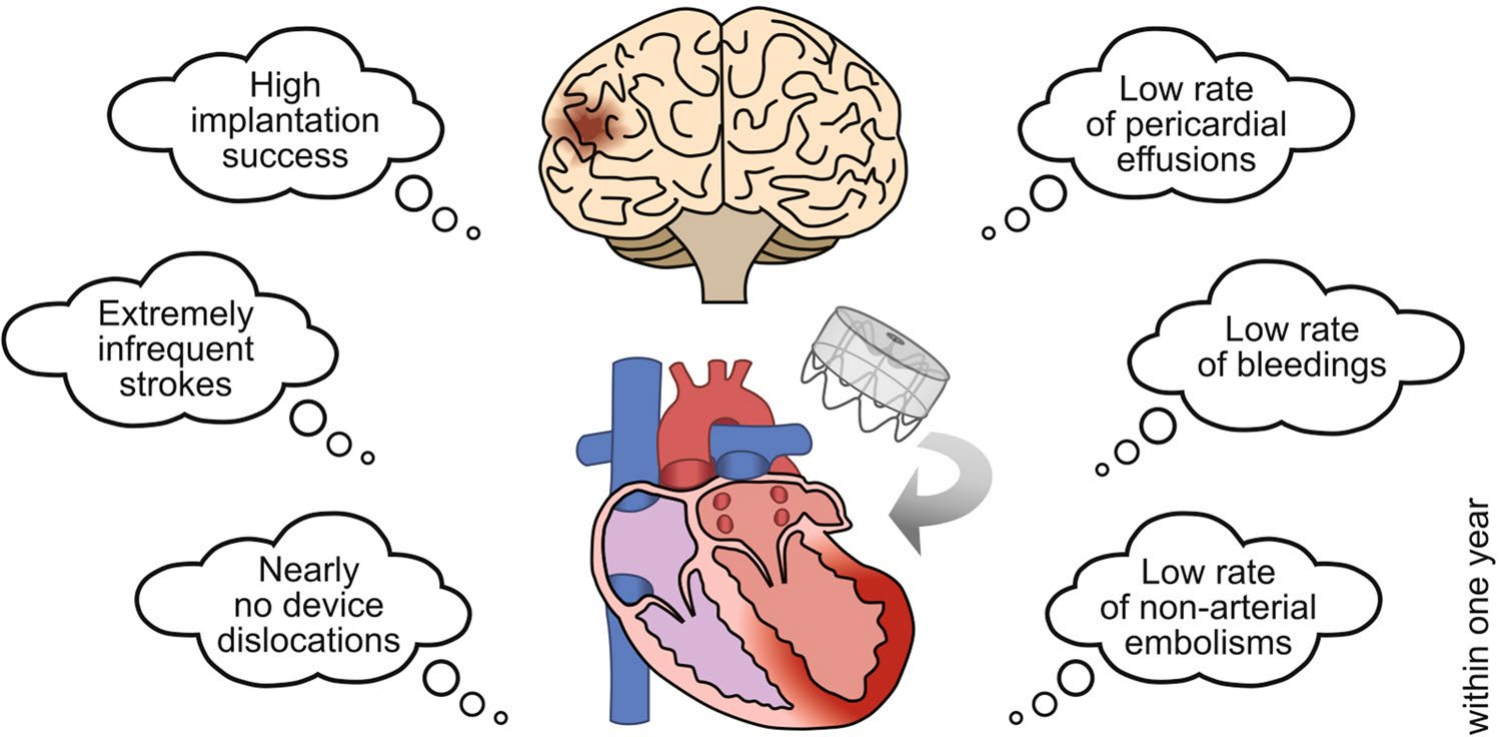

**Electronic supplementary material:**

The online version of this article (10.1007/s00392-020-01627-8) contains supplementary material, which is available to authorized users.

## Introduction

Atrial fibrillation (AF) is the most prevalent cardiac arrhythmia, with stroke and systemic embolization as prognostically relevant complications [1]. In AF patients with increased risk for thromboembolism, expressed by a CHA_2_DS_2_-VASc score ≥ 2, a prophylactic treatment is urgently needed, and routinely performed with oral anticoagulants (OAC). However, in some patients, these drugs are contraindicated for different reasons [1–3]. For these cases, the interventional left atrial appendage closure (LAAC) has evolved as the therapy of choice [1].

AF and heart failure due to a reduced left ventricular ejection fraction (LVEF) frequently coexist [4]. The prevalence of AF increases with the New York Heart Association (NYHA) functional class, so that nearly 5% of patients present with AF in NYHA functional class I, and almost 50% of patients in NYHA functional class IV [5]. AF patients with a severely reduced LVEF have a markedly increased risk for thromboembolic complications [6]. Therefore, there is a great need for effective thromboembolic prophylaxis especially in these patients. Data on periprocedural complications regarding cardiac interventions in patients with impaired LVEF are inconsistent [7, 8]. For example, patients with reduced LVEF are at a higher risk for device thrombosis [9]. After LAA closure, with regard to the long-term follow-up, the achievement of the primary efficacy endpoint was consistent for patients with LVEF ≥ 60% and those with impaired LVEF in the PROTECT-AF study [10]. However, in this study, patients with an LVEF < 30% were excluded by protocol, and the authors stated that this was due to the fact that the intervention in the LAA was naturally insufficient to prevent thromboembolism from the highly impaired left ventricle (LV). Nonetheless, patients with reduced LVEF are at risk of left atrial (LA) cavity thrombi [11]. A site-specific technique of thromboembolic prophylaxis with subsequent cessation of systemic anticoagulation might, therefore, be associated with an increased thromboembolic risk; yet, this is not officially specified as contraindication for this procedure.

The present subanalysis of the Left-Atrium-Appendage occluder Register—GErmany (LAARGE; ClinicalTrials.gov Identifier: NCT02230748) aimed to investigate procedural and one-year outcome of LAAC patients with and without LV dysfunction.

## Methods

### Enrollment and conduction of procedure

LAARGE is a prospective, multicenter real-world registry, which enrolled patients from 37 centers. It had been designed to compile a large database on daily practice of LAAC and clinical follow-up data. Recruitment started in July 2014, and ended in January 2016. Patients which were scheduled for an LAAC were consecutively included in the LAARGE database. Cases with started procedure and documented LVEF were included in the present subanalysis (as-treated analysis). The indication was set by the attending cardiologist in consideration of the dedicated guidelines [1], and the patients were treated according to the current recommendations and the manufacturer’s instructions with different post-market devices [12]. The postprocedural management including the antithrombotic treatment was at the discretion of the operating physician [13]. The study was carried out according to the principles of the 1975 Declaration of Helsinki, and was approved by the ethics committee of the Landesärztekammer Rheinland-Pfalz. Written informed consent to participate in the registry was obtained from all patients.

### Definition of reduced LVEF

LVEF dysfunction was defined as an LVEF 36–55% meaning a moderate reduction (mrLVEF), and ≤ 35% meaning a severe reduction (srLVEF).

### Data acquisition

Baseline characteristics, imaging, and procedural data as well as intra-hospital complications were reported by each center based on an electronic case report form. Adverse events and complications during the one-year follow-up were registered by the Stiftung Institut für Herzinfarktforschung (IHF) via a standardized phone interview with each patient, and by reports from the implantation centers. In the case of relevant complications, original medical records were reviewed by an event adjudication committee to allow similar assessment of all events. If patients could not be contacted, information was obtained from the residents’ registration offices.

### Outcome measures

Effectiveness was primarily assessed by the absence of all-cause death and stroke during follow-up, secondarily by the absence of systemic embolism and transient ischemic attacks (TIA). Intra-hospital complications including para-device leaks > 5 mm as well as severe and moderate bleedings, and device dislocations during follow-up constituted the safety outcome measure. Implantation success was defined as a stable device anchorage.

### Statistics

Statistical analyses were performed with SAS^®^ version 9.4 (SAS Institute, Cary, NC, USA). Continuous data are presented as means with standard deviation, or as medians with interquartile ranges (25th and 75th percentiles), categorical data as frequencies with group-related percentages. Trends across the patient groups were assessed by a Cochran–Armitage test regarding categorical variables, or by an exact Cochran–Armitage test in case of rare events, and by a Jonckheere–Terpstra test regarding metrical variables, as indicated in the tables. In addition, the group with srLVEF was compared with the rest using the Pearson chi-squared test, Fisher’s exact test, or Mann–Whitney–Wilcoxon test for categorical variables, rare events, and metrical variables, respectively. These statistics were based on the available cases.

One-year mortality after the implantation procedure, and the incidence of the combined event of all-cause death or non-fatal stroke were evaluated by means of survival analysis. Hazard ratios (HR) with 95% confidence intervals (CI) were estimated using Cox regression without adjustment, and adjusted for baseline risk factors significantly associated with LV function: age (linear), sex, coronary artery disease, peripheral arterial disease, chronic kidney disease, and diabetes mellitus. Expected yearly rates of non-fatal stroke were calculated from the individual CHA_2_DS_2_-VASc score, respectively [14]. The follow-up duration was defined as the time span from index discharge to the date of the follow-up contact. *p* values ≤ 0.05 (two-tailed) were considered significant.

## Results

### Baseline characteristics

619 patients with documented LVEF were included in this subanalysis. 344 (55.6%) revealed a preserved (p)LVEF, 225 (36.3%) an mrLVEF, and 50 (8.1%) an srLVEF (Table 1). Patients were predominantly older than 64 years of age (91.4%; *p* = NS). Among srLVEF patients male sex, coronary artery disease, and renal impairment were more prevalent (each *p* < 0.01). Both CHA_2_DS_2_-VASc (4.3 ± 1.5, 4.8 ± 1.6, and 5.1 ± 1.5, respectively; *p* < 0.001 for trend) and HAS-BLED score (3.7 ± 1.1, 4.1 ± 1.2 and 4.2 ± 1.0, respectively; *p* < 0.001 for trend) were the highest in the srLVEF group. Across all groups, the main indication was a prior bleeding event (80.0%; *p* = NS for trend).

**Table 1:** Baseline characteristics

	pLVEF	mrLVEF	srLVEF	*p* value*
Total cohort, *n* (% of all patients)	344 (55.6)	225 (36.3)	50 (8.1)	–
Male sex, *n* (%)	196 (57.0)	142 (63.1)	40 (80.0)	**0.002**^**#**^
Age [years], median (IQR)	76 (72; 80)	78 (73; 83)	77 (73; 81)	**0.019**
Body mass index [kg/m^2^], median (IQR)	26 (24; 30)	27 (24; 30)	27 (25; 29)	0.66
CHA_2_DS_2_-VASc score, mean ± SD	4.3 ± 1.5	4.8 ± 1.6	5.1 ± 1.5	** < 0.001**^**#**^
HAS-BLED score, mean ± SD	3.7 ± 1.1	4.1 ± 1.2	4.2 ± 1.0	** < 0.001**^**#**^
Arterial hypertension, *n *(%)	325 (94.5)	206 (91.6)	44 (88.0)	0.054
Diabetes mellitus, *n* (%)	103 (29.9)	87 (38.7)	22 (44.0)	**0.009**
Prior cerebrovascular event, each *n* (%)				
TIA	34 (9.9)	15 (6.7)	2 (4.0)	0.076
Stroke	73 (21.2)	49 (21.8)	9 (18.0)	0.77
Coronary heart disease, *n* (%)	121 (35.2)	129 (57.3)	36 (72.0)	** < 0.001**^**#**^
eGFR [MDRD], median (IQR)	65.4 (48.3; 81.5)	54.3 (36.4; 77.5)	49.1 (36.0; 79.7)	** < 0.001**^**#**^
Prior severe bleeding event, *n* (%)	128 (37.2)	94 (41.8)	25 (50.0)	0.067
Type of AF, each *n* (%)				
Paroxysmal	151 (43.9)	88 (39.1)	22 (44.0)	0.52
Persistent	59 (17.2)	42 (18.7)	9 (18.0)	0.71
Permanent	134 (39.0)	95 (42.2)	19 (38.0)	0.72
Indication for LAAC, each *n* (%)				
Prior bleeding	270 (78.5)	186 (82.7)	39 (78.0)	0.52
Prior cerebrovascular event despite anticoagulation	98 (28.5)	59 (26.2)	10 (20.0)	0.22
Adverse drug reaction	66 (19.2)	42 (18.7)	11 (22.0)	0.81
Labile INR	26 (7.6)	23 (10.2)	4 (8.0)	0.48
Incompliance with anticoagulation	19 (5.5)	12 (5.3)	2 (4.0)	0.71
Patient’s preference	97 (28.2)	52 (23.1)	11 (22.0)	0.15
Other reason	31 (9.0)	19 (8.4)	5 (10.0)	0.98
Medication at presentation, each *n* (%)				
Anticoagulation	201 (58.4)	138 (61.3)	36 (72.0)	0.093
Antiplatelet agent	110 (32.0)	83 (36.9)	24 (48.0)	**0.025**^**#**^

*AF *atrial fibrillation, *eGFR *estimated glomerular filtration rate, *INR *international normalized ratio, *IQR *interquartile range, *LAAC *left atrial appendage closure, *LVEF *left ventricular ejection fraction, *MDRD *modification of diet in renal disease, *SD* standard deviation, *TIA *transient ischemic attack

*Tested by Cochran–Armitage or Jonckheere–Terpstra test (*p* ≤ 0.05 is indicating a significant difference); ^#^significant difference between LVEF ≤ 35% and > 35% (*p* ≤ 0.05; tested by Pearson chi-squared or Mann–Whitney–Wilcoxon test)

The LA diameter increased with decreasing LVEF (*p* < 0.001 for trend; supplemental Table 1). No LAA morphology was predominant in any group.

### Procedural data and intra-hospital complications

Implantation success was achieved in 97.9%, and was independent from LVEF (*p* = NS for trend; Table 2). No peri-device leak > 5 mm was evident. Two interruptions were not related to LAA anatomy: in one patient, the transseptal sheath was destroyed intraprocedurally; the other patient developed an ST-segment elevation myocardial infarction. In an additional two patients, technical success was only achieved in a second procedure. There was no significant trend in the use of device types. 44.6% received a WATCHMAN™ device (Boston Scientific, Marlborough, MA, USA), 27.5% an AMPLATZER™ Cardiac Plug, and 24.9% an AMPLATZER™ Amulet™ (both Abbott, Chicago, IL, USA). Except from dose–area product, which increased with LVEF reduction (*p* = 0.004 for trend), procedural parameters were statistically indifferent across all groups.

**Table 2:** Procedural data

	pLVEF	mrLVEF	srLVEF	*p* value*
Total cohort, *n* (% of all patients)	344 (55.6)	225 (36.3)	50 (8.1)	–
Successful implantation, *n* (%)	336 (97.7)	221 (98.2)	49 (98.0)	0.72
Number of implantation attempts, mean ± SD	1.7 ± 1.3	1.6 ± 1.2	1.5 ± 0.9	0.96
Peri-device leak, each *n* (%)	17 (5.1)	14 (6.3)	1 (2.0)	0.91
< 3 mm	13	10	1	
3–5 mm	4	4	0	
> 5 mm	0	0	0	
Type of LAAC device, each *n* (%)				
WATCHMAN™	164 (47.7)	90 (40.0)	22 (44.0)	0.16
AMPLATZER™ Cardiac Plug	93 (27.0)	59 (26.2)	18 (36.0)	0.43
AMPLATZER™ Amulet™	75 (21.8)	69 (30.7)	10 (20.0)	0.25
Other device	12 (3.5)	7 (3.1)	0 (0.0)	0.36
Total duration [min], median (IQR)	60 (45; 80)	57 (41; 76)	60 (43; 81)	0.32
Fluoroscopy time [min], median (IQR)	11 (7; 15)	10 (7; 15)	9 (6; 15)	0.36
Dose–area product [Gy*cm^2^], median (IQR)	1821 (638; 4011)	2303 (1027; 4226)	3187 (1279; 5784)	**0.004**^**#**^
Sedation type, each *n* (%)				
Conscious sedation	287 (83.4)	193 (85.8)	39 (78.0)	0.85
General anesthesia	40 (11.6)	21 (9.3)	11 (22.0)	0.30
Other	10 (2.9)	7 (3.1)	0 (0.0)	0.36
None	7 (2.0)	4 (1.8)	0 (0.0)	0.40

*IQR* interquartile range, *LAAC *left atrial appendage closure, *LVEF *left ventricular ejection fraction, *SD *standard deviation

*Tested by Cochran–Armitage or Jonckheere–Terpstra test (*p* ≤ 0.05 is indicating a significant difference); ^#^significant difference between LVEF ≤ 35% and > 35% (*p* ≤ 0.05; tested by Pearson chi-squared or Mann–Whitney–Wilcoxon test)

Intra-hospital complications, especially major adverse cardiac and cerebrovascular events (MACCE), and other major complications were throughout infrequent, and showed a balanced distribution (Table 3). Two patients died due to an unknown or a cardiovascular reason other than the implant procedure, respectively, and the cases of death were hence classified as adverse events. Eight dislodged devices could be snared by a catheter (each *p* = NS for trend). Pursuant to this low number of complications, the period of hospitalization was generally short (median 2 days; *p* = NS for trend).

**Table 3:** Intra-hospital outcome

	pLVEF	mrLVEF	srLVEF	*p* value*
Total cohort, *n* (% of all patients)	344 (55.6)	225 (36.3)	50 (8.1)	–
MACCE, *n* (%)	1 (0.3)	2 (0.9)	0 (0.0)	1.00
Death, *n* (%)	1 (0.3)	1 (0.4)	0 (0.0)	1.00
Myocardial infarction, *n* (%)	0 (0.0)	1 (0.4)	0 (0.0)	1.00
Stroke, *n* (%)	0 (0.0)	1 (0.4)	0 (0.0)	1.00
Other major complications, *n* (%)	14 (4.1)	11 (4.9)	1 (2.0)	0.88
Severe bleeding, *n* (%)	3 (0.9)	3 (1.3)	1 (2.0)	0.55
AV fistula or pseudoaneurysm, *n* (%)	3 (0.9)	3 (1.3)	0 (0.0)	1.00
Pericardial effusion requiring action, each *n* (%)				
Surgery	1 (0.3)	1 (0.4)	0 (0.0)	1.00
Intervention	7 (2.0)	5 (2.2)	1 (2.0)	1.00
Device dislodgement requiring action, each *n* (%)	1 (0.3)	1 (0.4)	0 (0.0)	1.00
Moderate complications, *n* (%)	32 (9.3)	21 (9.3)	6 (12.0)	0.67
Moderate bleeding, *n* (%)	6 (1.7)	3 (1.3)	2 (4.0)	0.64
TIA, *n* (%)	0 (0.0)	0 (0.0)	0 (0.0)	–
Successful cardiopulmonary resuscitation, *n* (%)	2 (0.6)	1 (0.4)	0 (0.0)	0.70
Access site infection, *n* (%)	1 (0.3)	0 (0.0)	0 (0.0)	0.64
Pericardial effusion with conservative treatment, *n* (%)	5 (1.5)	6 (2.7)	0 (0.0)	1.00
Device dislodgement handled by immediate retraction, *n* (%)	4 (1.2)	2 (0.9)	0 (0.0)	0.55

*AV *arteriovenous, *LVEF *left ventricular ejection fraction, *MACCE *major adverse cardiac and cerebrovascular events, *TIA *transient ischemic attack

*Tested by exact Cochran–Armitage test (*p* ≤ 0.05 is indicating a significant difference)

12.2% of patients received anticoagulants at hospital discharge (*p* = NS for trend; supplemental Table 2). Patients who received dual antiplatelet agents were prescribed this therapy for 3–6 months after the procedure (*p* = NS for trend), while the majority of these patients (59.0%) stayed on this medication for only 3 months.

### One-year follow-up

**Fig. 1:**
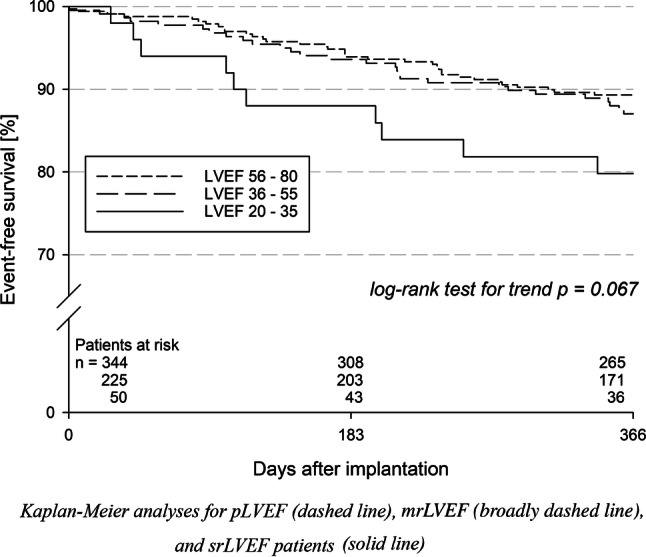
Freedom from all-cause death and non-fatal stroke after left atrial appendage closure

For 602 patients (97.6% of all patients who had been discharged alive), a follow-up was documented (*p* = NS for trend; Table 4). The combined primary effectiveness outcome measure, i.e., absence of all-cause death and non-fatal stroke, was reached in 87.7% of patients within 365 days after the procedure (*p* = 0.067 for trend; Fig. 1). In a simple Cox regression model, an increased incidence of all-cause death or stroke in the srLVEF group compared to the pLVEF group was evident. However, after adjustment for age, sex, chronic kidney disease, coronary artery disease, peripheral arterial disease, and diabetes mellitus, the effect of LVEF on the combined effectiveness outcome measure did not reach statistical significance any more (Table 5). In contrast, chronic kidney disease (HR: 3.18; 95% CI: 1.90–5.32; *p* < 0.001), coronary artery disease (HR: 1.86; 95% CI: 1.03–3.33; *p* = 0.038), and peripheral arterial disease (HR: 1.72; 95% CI: 1.04–2.86; *p* = 0.035) were identified as independent predictors. Thromboembolic events were generally rare: non-fatal ischemic strokes occurred in 0.6% of patients, TIA in 0.4%, and systemic embolisms in 0.2% (each *p* = NS for trend). This was despite the fact that only 5.9% of patients who persisted on OAC after 1 year (*p* = NS for trend), whereby the distribution of anticoagulant agents did not differ between the groups (supplemental Table 2). In one pLVEF patient with a WATCHMAN™ device, a device-related thrombus (DRT) was detected after 1.5 months by transesophageal echocardiography (information reported for 217 patients from 20 centers, *p* = NS between LVEF groups; median time point 1.3 (2.7; 5.7) months). The DRT could be resolved by resumption of phenprocoumon for three months. No further thromboembolic event was registered in this patient during follow-up.

**Table 4:** One-year follow-up

	pLVEF	mrLVEF	srLVEF	*p* value*
Discharged alive, *n*	343	224	50	
Information on vital status obtained, *n* (%)	331 (96.5)	221 (98.7)	50 (100.0)	0.064
Death within 365 days, *n* (% of patients with documented vital status)	33 (10.0)	24 (10.9)	10 (20.0)	0.096^**#**^
Events in survivors of follow-up (365 days)				
Surviving patients with detailed follow-up information, *n*	282	183	32	
Major adverse events				
Stroke, *n* (%)	1 (0.4)	2 (1.1)	0 (0.0)	0.64
TIA, *n* (%)	1 (0.4)	1 (0.5)	0 (0.0)	1.00
Systemic embolism, *n* (%)	1 (0.4)	0 (0.0)	0 (0.0)	1.00
Device dislodgement requiring action, each *n* (%)				
Surgery	2 (0.7)	0 (0.0)	0 (0.0)	0.58
Additional intervention	2 (0.7)	0 (0.0)	0 (0.0)	0.58
Pericardial effusion requiring action, *n* (%)	1 (0.4)	0 (0.0)	0 (0.0)	1.00
Severe groin complication, *n* (%)	1 (0.4)	1 (0.5)	1 (3.1)	0.17
Pulmonary embolism, *n* (%)	2 (0.7)	3 (1.6)	1 (3.1)	0.19
Severe bleeding, *n* (%)	2 (0.7)	0 (0.0)	1 (3.1)	0.64
Moderate adverse events				
Deep vein thrombosis, *n* (%)	0 (0.0)	2 (1.1)	0 (0.0)	0.26
Moderate bleeding, *n* (%)	10 (3.5)	8 (4.4)	2 (6.3)	0.46
Rehospitalization, *n* (%)°	94 (37.2)	69 (42.6)	10 (33.3)	0.70

*LVEF *left-ventricular ejection fraction, *TIA *transient ischemic attack

*Tested by exact Cochran–Armitage (events) or asymptotic Cochran–Armitage test (*p* < 0.05 is indicating a significant difference); ^#^significant difference between LVEF ≤ 35% and > 35% (*p* < 0.05; tested by Fisher’s exact test); °data available for 253 (pLVEF), 162 (mrLVEF), and 30 (srLVEF) patients, respectively

**Table 5:** Cox regression analysis for the primary effectiveness outcome measure

	Hazard ratio	95% CI	*p* value
Unadjusted effects			
LVEF ≤ 35%	2.04	1.01–4.11	0.138
LVEF 36–55%	1.22	0.74–2.01	
Adjusted effects*			
LVEF ≤ 35%	1.09	0.53–2.27	0.690
LVEF 36–55%	0.84	0.50–1.39	

*CI *confidence interval, *LVEF *left ventricular ejection fraction

*Adjusted for age (linear), sex, chronic kidney disease, coronary artery disease, peripheral arterial disease, and diabetes mellitus; *p* ≤ 0.05 indicates significant difference

Concerning the safety outcome measures, the rates of device dislocations (0.8%), severe (0.6%), and moderate (4.0%) bleedings were low, and independent of LVEF. Three patients needed blood transfusions. Moreover, thromboembolisms in the venous system were rare events (1.6%, *p* = NS for trend).

## Discussion

The present subanalysis of the multicenter LAARGE registry revealed that the effectiveness of stroke prevention by LAAC is high and persistent in AF patients with LV myocardial impairment. Despite srLVEF patients being prone to a pre-existing relevantly increased thromboembolic risk [6], this finding was also applicable to this subgroup of patients.

Though prima facie, the combined event rate of all-cause deaths and non-fatal strokes showed a trend towards a higher incidence with LVEF reduction, this only reflects the expectable accompanying increase in cardiovascular comorbidity. Consistently, LVEF impairment was associated with an increase in CHA_2_DS_2_-VASc score (4.3, 4.8 and 5.1, respectively). It is, therefore, not remarkable that all-cause deaths were accountable for at least 95.7% of the events in the primary outcome measure. After adjustment for relevant confounders, a significant difference between the LVEF groups was no longer evident. Moreover, the observed annual stroke rates (0.4% in pLVEF, 1.1% in mrLVEF, and 0% in srLVEF patients, respectively) were substantially reduced, especially when compared to the annual rates estimated by the CHA_2_DS_2_-VASc score (5.4% in pLVEF, 6.2% in mrLVEF, and 6.7% in srLVEF patients, respectively) [14]. TIAs (0.4%) and systemic embolisms (0.2%) were absolutely infrequent across all groups.

Such a low rate of thromboembolism was reached although patients with severely impaired LV myocardium were not anticoagulated to a higher extent after the procedure, neither did they present with a higher percentage of prior strokes at baseline. These findings could extend the evidence that OAC is dispensable in those patients with regard to LV thromboembolism [10, 15]. Though one must bear in mind that the present results are limited to one year, and thromboembolic complications might occur later on.

Across all groups, implantation success (97.9%), i.e., stable device anchorage without a relevant peri-device leak, was very high compared to the initial trials on LAAC [16, 17], irrespective of a diversified prior experience of the participating operators. Similar technical feasibility was also reflected by a resembling procedural duration and fluoroscopy time. Unlike in other cardiac interventions [7, 18], LAAC was not accompanied by an elevated rate of periprocedural complications or deaths in patients with reduced LVEF. Especially MACCE, but also other major complications were not only infrequent (0.5 and 4.2%, respectively), but also comparably distributed between the LVEF groups, and the frequencies were in line with other recently published data [19, 20]. These low complication rates might be due to leaving out the high-pressure LV system, or the ventricular myocardium from the intervention.

Together with a consistently low rate of periprocedural complications, major bleedings (0.6%) and non-arterial thromboembolic events (1.6%) were infrequent during follow-up, and constituted for adequate safety.

For the first time, the well-established LAAC intervention was shown to not only come along with high procedural success across all stages of LVEF, but also to guarantee excellent stroke prophylaxis irrespective of LV myocardial impairment. In summary, there was no evidence which justifies excluding patients with reduced EF from this intervention.

### Limitations

These analyses were based on data of a real-world registry with some inherent limitations. Respecting the observational character of this registry, conduction of the intervention was not influenced by the study investigators, and was based on the operators’ discretions as well as the manufacturers’ recommendations. This individualized decision algorithm might have influenced the outcome measures, but surely reflects the clinical practice. There is no information about the implantation volume per center and per operator during the study period. Adverse events during follow-up were partly self-reported, which might have lessened the detected numbers. The individual reason for a maintained anticoagulation after the procedure could not be extracted from the original data. In addition, the follow-up was limited to one year. However, despite the limitations of this observational registry, it is serving as a data source for a little studied topic.

## Conclusion

Neither the procedural success nor the periprocedural complication rate was influenced by LV systolic function. Despite a diversified risk profile at baseline, annual rates of non-fatal strokes and major bleedings were low across all groups, which meant a substantial risk reduction as compared to the estimated risks. LAAC, therefore, appeared as a feasible intervention in AF patients with reduced LVEF.

## Electronic supplementary material

Below is the link to the electronic supplementary material.

Supplementary file1 (DOCX 18 kb)

## Abbreviations

AF: Atrial fibrillation
CI: Confidence interval
DRT: Device-related thrombus
HR: Hazard ratio
IHF: Stiftung Institut für Herzinfarktforschung
LA(A): Left atrial (appendage)
LAAC: Left atrial appendage closure
LAARGE: Left-Atrium-Appendage occluder Register—GErmany
(LV)EF: (Left ventricular) ejection fraction
MACCE: Major adverse cardiac and cerebrovascular events
OAC: Oral anticoagulation
NS: Not significant
TIA: Transient ischemic attack
