# Supplementary material for: Left atrial appendage closure in patients with a reduced left ventricular ejection fraction: results from the multicenter German LAARGE registry
Source: Clin Res Cardiol. 2020 Mar 31;109(11):1333–41. doi: 10.1007/s00392-020-01627-8 (PMC7588387; doi:10.1007/s00392-020-01627-8)
Supplement: Supplementary file 1 — Supplementary file1 (DOCX 18 kb) [file 392_2020_1627_MOESM1_ESM.docx]

| **Supplemental table 1: Preinterventional imaging data** | | | | |
| --- | --- | --- | --- | --- |
|  | **pLVEF** | **mrLVEF** | **srLVEF** | **p value** |
| **Total cohort, n (% of all patients)** | 336 (57.4) | 202 (34.5) | 47 (8.0) | --- |
| **LVEF [%], median (IQR)** | 60 (60; 65) | 50 (45; 55) | 30 (25; 35) | **<0.001^#^** |
| **LA diameter [mm], median (IQR)** | 47 (43; 51) | 48 (45; 52) | 50 (48; 55) | **<0.001^#^** |
| **LAA ostial diameter [mm], median (IQR)**   - **0°** - **45°** - **90°** - **135°** | 20 (18; 22)  20 (17; 22)  20 (17; 22)  20 (18; 22) | 20 (18; 22)  20 (18; 23)  20 (18; 23)  20 (18; 23) | 21 (19; 23)  21 (19; 22)  21 (18; 24)  21 (19; 23) | 0.16  **0.008**  0.064  0.080 |
| **LAA morphology, each n (%)**   - **cactus** - **cauliflower** - **chicken wing** - **windsock** - **atypical** | 32 (9.5)  48 (14.3)  155 (46.1)  54 (16.1)  47 (14.0) | 14 (6.9)  35 (17.3)  85 (42.1)  27 (13.4)  41 (20.3) | 6 (12.8)  7 (14.9)  19 (40.4)  8 (17.0)  7 (14.9) | 0.97  0.51  0.34  0.75  0.30 |
| **Number of lobi, each n (%)**   - **1** - **2** - **>2** | 195 (58.0)  125 (37.2)  16 (4.8) | 99 (49.0)  87 (43.1)  16 (7.9) | 23 (48.9)  16 (34.0)  8 (17.0) | **0.015** |
| **LA sludge, n (%)**  **LA thrombus, n (%)** | 38 (11.3)  0 (0.0) | 37 (18.3)  1 (0.5) | 12 (25.5)  0 (0.0) | **0.002^#^**  0.45 |
| **LAA thrombus, n (%)** | 1 (0.3) | 2 (1.0) | 1 (2.1) | 0.13 |
| * tested by Cochran-Armitage or Jonckheere-Terpstra test (p≤0.05 is indicating a significant difference); # is indicating a significant difference between LVEF ≤35% and >35% (p≤0.05; tested by Pearson chi-squared or Mann-Whitney-Wilcoxon test); IQR = interquartile range; LA = left atrial; LAA = left atrial appendage; LVEF = left ventricular ejection fraction | | | | |

| **Supplemental table 2: Antithrombotic therapy** | | | | |
| --- | --- | --- | --- | --- |
|  | **pLVEF** | **mrLVEF** | **srLVEF** | **p value*** |
| **Antithrombotic therapy at discharge, each n (% of all patients discharged alive)**   - **DAPT** - **anticoagulation** | 292 (85.1)  44 (12.8) | 201 (89.7)  23 (10.3) | 44 (88.0)  8 (16.0) | 0.19  0.94 |
| **Antithrombotic therapy after one year, each n (%)^†^**   - **antiplatelet agents** - **anticoagulation** | 232 (82.9)  15 (5.4) | 158 (86.8)  11 (6.0) | 28 (87.5)  3 (9.4) | 0.24  0.43 |
| * tested by Cochran-Armitage test (p<0.05 is indicating a significant difference); † data available for 280 (pLVEF), 182 (mrLVEF), and 32 (srLVEF) patients, respectively; DAPT = dual antiplatelet therapy; LVEF = left ventricular ejection fraction | | | | |
